# Effects of PM_10_ on mortality in pure COPD and asthma-COPD overlap: difference in exposure duration, gender, and smoking status

**DOI:** 10.1038/s41598-020-59246-2

**Published:** 2020-02-12

**Authors:** Yu Min Lee, Jin Hwa Lee, Hawn-Cheol Kim, Eunhee Ha

**Affiliations:** 1 Department of Occupational and Environmental Medicine, Ewha Womans University College of Medicine, Seoul, Republic of Korea; 2 Division of Pulmonary and Critical Care Medicine, Department of Internal Medicine, Ewha Womans University College of Medicine, Seoul, Republic of Korea; 3 Department of Occupational and Environmental Medicine, Inha University School of Medicine, Incheon, Republic of Korea

**Keywords:** Environmental impact, Epidemiology

## Abstract

We investigated the effects of particulate matter (PM) on mortality in patients diagnosed with asthma-COPD overlap (ACO) or ‘pure COPD’. Subjects from the National Health Insurance Service–National Sample Cohort of Korea, who were aged 40 years or above and had newly diagnosed COPD since 2009 were selected. Finally, 6,313 patients were enrolled and divided into ‘pure COPD’ and ACO groups. Average PM_10_ exposure data were obtained using Kriging interpolation from 2001 to 2013. Hazard ratios(HR) were estimated using a time-varying Cox regression model. Exposure to PM_10_ for 1, 3, and 6 months was associated with an increase in non-accidental mortality in the entire COPD group, especially the ACO group. When a stratified analysis of 3-month exposure was performed by sex, the highest HR was found in women with ACO (HR = 1.153; 95% confidence intervals [CI]: 1.121, 1.185). A stratified analysis according to smoking status showed that ACO patients had the highest HR among never smokers (HR = 1.151; 95% CI; 1.124, 1.178). Average exposure to PM_10_ was associated with non-accidental mortality in patients with COPD, especially those diagnosed with ACO. In addition, the adverse effects of PM_10_ exposure are more severe in women and never-smokers.

## Introduction

Ambient particulate matter (PM) is a mixture of thousands of components. When inhaled or otherwise entered the human body, the PM can deposit in the alveoli of the lungs and trigger local inflammation, which in turn can lead to systemic inflammation^1^. Previous studies demonstrated that compared with direct bronchodilators, anti-inflammatory medications can be used to ameliorate the asthmatic symptoms associated with PM with greater efficacy^2^. Direct experimental evidence of inflammation induced by PM, and modulating effects of inflammation-associated genes and pathways, have also been reported. PM is often categorized based on the aerodynamic diameter of the particles. The PM_10_ used in our study is particulate matter with a diameter of 10 micrometers or less. Previous studies have reported that substances such as changes in fraction of exhaled nitric oxide, exhaled hydrogen sulfide levels, and IL-8 concentrations in the nasal lavage fluid as respiratory inflammatory biomarkers related to particular matters^3^. Meanwhile, chronic obstructive pulmonary disease (COPD) is characterized by chronic airway inflammation, which may coexist with or without asthma. Airway inflammation affects the pathogenesis of both COPD and asthma; however inflammatory cells detected in both diseases and their prognosis differ^4,5^. Patients who were previously known to exhibit severe asthma and did not respond effectively to asthma medication, or patients with COPD who underwent frequent exacerbations associated with airway hyper-responsiveness represent case of asthma and COPD overlap (ACO)^6^. Therefore, patients with ACO are thought to be susceptible to PM_10_ because COPD and asthma exhibit overlapping features and are more frequently exacerbated. Many studies have shown that patients with ACO were more likely to manifest higher levels of comorbidities than patients diagnosed with only COPD^7^. An estimated 3.1 million deaths are attributed to PM exposure, accounting for 3.1% of global disability-adjusted life years lost worldwide in 2010^8^. Previous studies focused on the effect of PM on mortality rates of patients with COPD, not ACO. The purpose of this study was to evaluate the effects of mortality associated with average PM_10_ exposure (1, 3, 6, and 12 months) for short- and long-term periods in COPD patients. The study analyzed the differences in mortality between ‘pure COPD’ and ACO associated with ambient PM exposure, and identified the subgroups within the ACO group who were more vulnerable to PM exposure.

## Materials and Methods

### Source of data and study design

**Figure 1:**
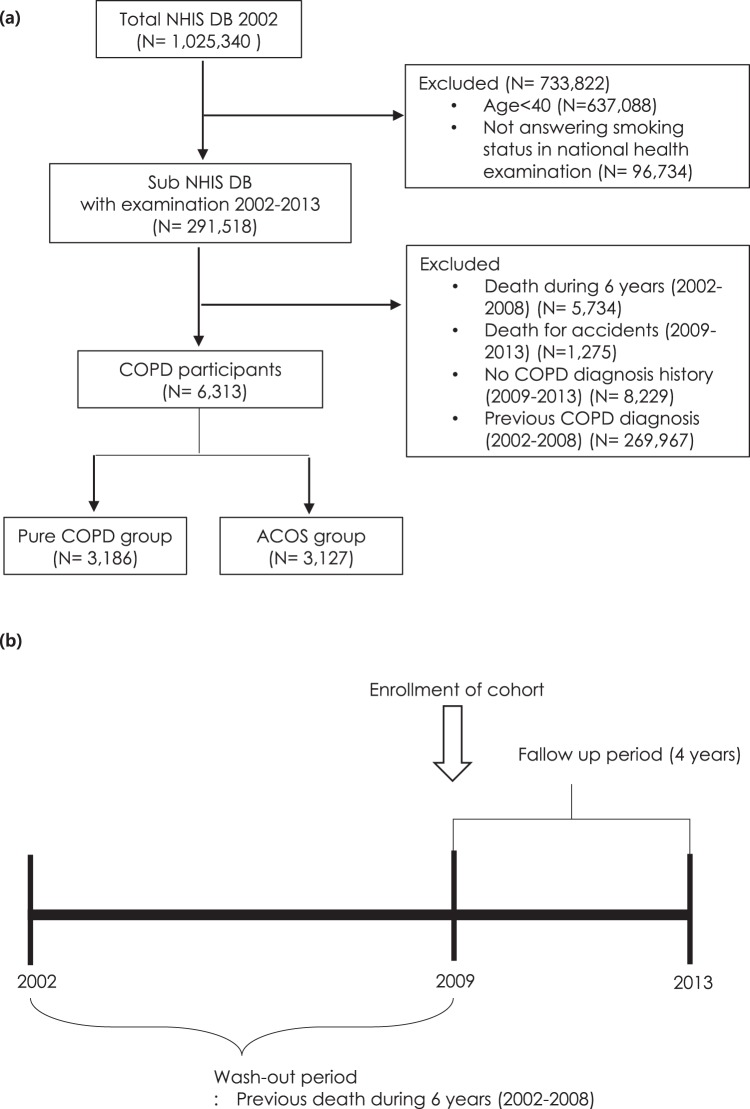
Study design (**a**) Flow diagram of the study design (**b**) Study design over time. NHIS DB = National Health Insurance Service database. COPD = chronic obstructive lung disease.

We used the National Health Insurance Service–National Sample Cohort (NHIS-NSC) database, a population-based cohort from 2002 to 2013, established by the NHIS in South Korea^9^. A sample population of 1,025,340 subjects was randomly selected and followed up until 2013. The NHIS-NSC contains the results of biannual health checkups and health care utilization. The enrolled population included, subjects aged 40 to 89 years (N = 291,518) who responded to a questionnaire, especially concerning a smoking habit. We sought individuals who had not been diagnosed with COPD prior to entering the cohort and also intended to use the data monitored from 2008 on which the reliability of the exposure data was secured. Therefore, the study subjects included individuals who were newly diagnosed with COPD based on the International Code of Diseases (ICD)-10 (J42.x-44.x except J430) from January 1, 2009 to December 31, 2013, and who were prescribed one or more COPD medications at least twice per year [long-acting muscarinic antagonist (LAMA), long-acting beta-2 agonist (LABA), inhaled corticosteroids (ICS), ICS plus LABA (ICS + LABA), short-acting muscarinic antagonist (SAMA), short-acting beta-2 agonist (SABA), or theophylline]^10^. During 2002–2008, patients diagnosed with COPD or who died for any reason, were excluded. Also excluded were those who were not diagnosed with COPD or died in an accident between 2009 and 2013. Finally, subjects with COPD were divided into a pure COPD group and an ACO group. The ACO group was defined by the criteria for COPD and asthma diagnosis. Asthma was defined by the J45.x or J46 criteria based on ICD-10 and treatment with one or more asthma medications at least twice per year from January 1, 2009 to December 31, 2013 [LAMA, LABA, ICS, ICS + LABA, SAMA, SABA, theophylline, leukotriene antagonist (LTRA), systemic corticosteroids, or systemic beta agonist]^10^ (Fig. 1).

The study was approved by the institutional review board of Ewha Womans University Mokdong Hospital, Seoul, Republic of Korea (IRB number: EUMC 2018-04-061). This study was performed in accordance with the relevant guidelines and regulations. Also the need for participants’ informed consent was waived by the ethics review board.

### Measurement of particular matter 10 (PM_10_) exposure

**Figure 2:**
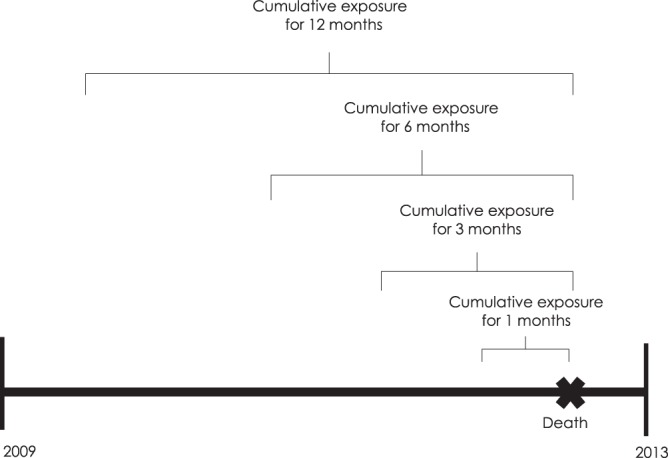
Particular matter (PM)10 exposure model.

The exposure level of PM_10_ at each subject’s residence was predicted using the Kriging modeling method. Kriging modeling was conducted between January 1, 2001 and December 31, 2013, based on daily exposure data of PM_10_ measured by beta-ray absorption methods at 38 national air monitoring centers located at three regions in Korea. The monthly pollutant levels for each place (place unit: dong) for PM_10_ were predicted based on the levels recorded on the monitors. A method known as ordinary block kriging, was conducted using the Geostatistical Analyst extension of ArcGIS (ArcMap, version 9.0; ESRI Inc., Redlands, WA, USA) with 0.170 km × 0.170 km grids to partition each dong and each month. Each block kriging was predicted used kriging with a regular grid and the values within each block were averaged. The quality of the predicted values was evaluated using cross-validation. The technique was repeated to remove each monitoring station one at a time. In addition, the concentration at each missing station (removed earlier) was estimated using the concentration levels detected on the other monitors. The observed concentrations at the monitoring sites were compared with the values predicted by kriging, as described previously^11^. Cohort data used in this study included patient address, which was matched with PM_10_ monitoring site address to predict the patient’s PM_10_ concentration. To determine the average effect of PM_10_ exposure on mortality in phases, the average exposures for PM_10_ were analyzed based on an average of 12 months, 6 months, 3 months and 1 month before the event (Fig. 2).

### Statistical analysis

Descriptive statistics of subjects are expressed as a number (%) or mean and standard deviation. We used a time-varying Cox regression model, with calendar time as the underlying time scale, to estimate the adjusted hazard ratio (aHR) for non-traumatic mortality^12,13^. All person-months were followed up until non-traumatic death or the end of 2013, whichever came first. To select covariates for inclusion in adjusted models, we performed a literature search in order to identify the risk factors associated with PM_10_ exposure, COPD, ACO, or non-traumatic death [sex, age, smoking status, body mass index, Charlson Comorbidity Index (CCI), and household income levels]. We then conducted subgroup analyses stratified by sex and smoking status, which are strong confounders in patients with pure COPD and ACO. All statistical analyses were carried out using SAS statistical software version 9.4 (SAS Institute Inc., Cary, NC, USA).

## Results

### Characteristics of subjects and PM_10_ distribution

The baseline characteristics of a total of 6,313 subjects are presented in Table 1. The pure COPD group constituted 50.5% of the total number of subjects and the rest belonged to the ACO group. The mean follow-up period of the study subjects was 4.9 years and 53.6% were men. The average PM_10_ exposures (1, 3, 6, and 12 months) did not differ between pure COPD and ACO. The 3-month values were the lowest in each group.

**Table 1:** Baseline characteristics of study subjects.

Variable	Total COPD	Pure COPD	ACO	P-value
	6,313	3,186 (50.5)	3,127 (49.5)	$0{.4577}^{a}$
Follow-up period (years)	4.9 ± 0.5	4.9 ± 0.5	4.9 ± 0.5	$0{.7633}^{b}$
Average PM_10_ exposure (10 μg/m^3^)				
12 months	48.2 ± 5.7	48.1 ± 5.7	48.2 ± 5.7	${0.4160}^{b}$
6 months	41.2 ± 5.1	41.1 ± 5.2	41.2 ± 5.1	${0.2742}^{b}$
3 months	39.5 ± 5.2	39.4 ± 5.3	39.5 ± 5.2	${0.4531}^{b}$
1 month	46.4 ± 6.1	46.5 ± 6.0	46.4 ± 6.2	${0.7554}^{b}$
Sex				$0{.0064}^{a}$
Men	3386 (53.6)	1763 (55.3)	1623 (51.9)	
Women	2927 (46.4)	1423 (44.7)	1504 (48.1)	
Age groups				$$ < 0{.0001}^{a}$$
40~49 years	1478 (23.4)	835 (26.2)	643 (20.6)	
50~59 years	1971 (31.2)	1007 (31.6)	964 (30.8)	
60~69 years	2144 (34.0)	1025 (32.2)	1119 (35.8)	
70 years +	720 (11.4)	319 (10.0)	401 (12.8)	
Smoking status				
Never-smokers	3348 (53.0)	1678 (52.7)	1670 (53.4)	$0{.6121}^{a}$
Former smokers	967 (15.3)	502 (15.8)	465 (14.9)	
Current smokers	1998 (31.7)	1006 (31.6)	992 (31.7)	
Body mass index (kg/m^2^)	23.9 ± 3.3	23.8 ± 3.3	24.0 ± 3.4	$0{.0025}^{b}$
Charlson Comorbidity Index	2. 1 ± 1.6	1.9 ± 1.6	2.2 ± 1.5	$$ < 0{.0001}^{b}$$
Income levels				$0{.1102}^{a}$
0	180 (2.9)	89 (2.8)	91 (2.9)	
0–10%	540 (8.6)	249 (7.8)	291 (9.3)	
10–20%	440 (7.0)	218 (6.8)	222 (7.1)	
20–30%	462 (7.3)	221 (6.9)	241 (7.7)	
30–40%	508 (8.1)	245 (7.7)	263 (8.4)	
40–50%	552 (8.7)	268 (8.4)	284 (9.1)	
50–60%	571 (9.0)	286 (9.0)	285 (9.1)	
60–70%	678 (10.7)	349 (11.0)	329 (10.5)	
70–80%	687 (10.9)	361 (11.3)	326 (10.4)	
80–90%	828 (13.1)	425 (13.3)	403 (12.9)	
90–100%	867 (13.7)	475 (14.9)	392 (12.5)	

p-value^*a*^ for Chi square. p-value^*b*^ for T test.

Data is presented as mean ± SD or number (%).

### Total non-accidental mortality analysis

**Figure 3:**
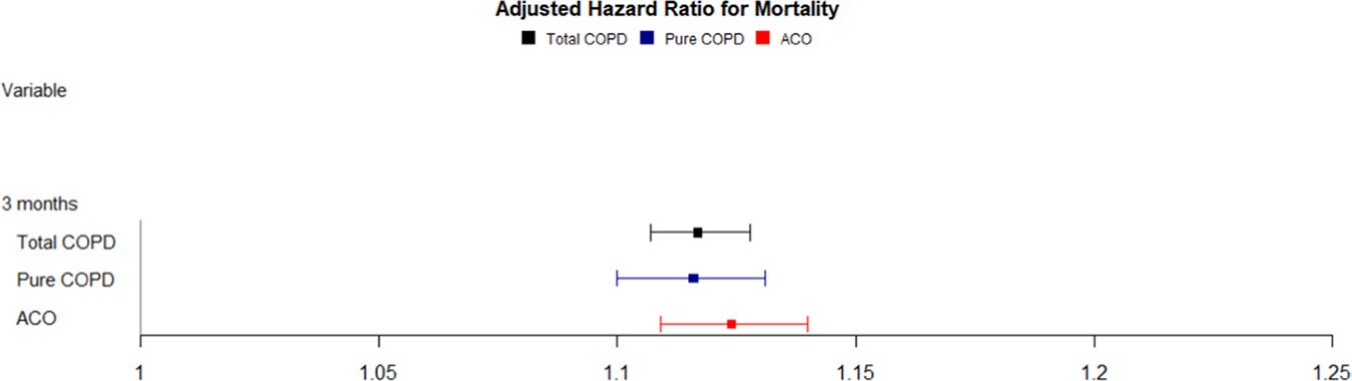
Effect of particular matter on mortality in the whole cohort over a 3-month PM_10_ exposure. COPD = chronic obstructive lung disease, ACO = asthma-COPD overlap.

In the Cox regression analysis, aHRs for each 10-μg/m^3^ increment in PM_10_ after a 12-month average exposure was 1.011(95% CI, 0.994–1.027) in total COPD patients, which was borderline significant statistically. When mortality was analyzed for exposure in the recent 6 months, aHRs were 1.124 (95% CI, 1.106–1.142) for the pure COPD group, 1.125 (95% CI, 1.108–1.143) for the ACO group, and 1.123 (95% CI, 1.111–1.135) for all COPD subjects, all of which were statistically significant. After exposure for the last 3 months, aHRs were 1.116 (95% CI, 1.100–1.131) in the pure COPD group and 1.124 (95% CI, 1.109–1.140) in the ACO group. Based on the shortest period of exposure, 1-month, aHRs were 1.017 (95% CI, 0.998–1.036) for the pure COPD group and 1.026 (95% CI, 1.007–1.046) for the ACO group (Table 2, Fig. 3). Mortality associated with the average 3-month exposure is shown in Fig. 3. The 3-month exposure-associated mortality showed the largest difference in statistically significant mortality associated with PM_10_ exposure.

**Table 2:** Hazard ratios for death according to PM_10_ exposure duration.

	N (%)	HR	95% CI		aHR	95% CI	
**12 months**							
Total COPD	6,313	1.018	1.001	1.035	1.011	0.994	1.027
Pure COPD	3,186 (50.47)	1.015	0.992	1.040	1.004	0.981	1.028
ACO	3,127 (49.53)	1.020	0.997	1.044	1.015	0.993	1.039
**6 months**							
Total COPD	6,313	1.179	1.167	1.190	1.123	1.111	1.135
Pure COPD	3,186 (50.47)	1.186	1.170	1.202	1.124	1.106	1.142
ACO	3,127 (49.53)	1.171	1.155	1.188	1.125	1.108	1.143
**3 months**							
Total COPD	6,313	1.166	1.156	1.176	1.117	1.107	1.128
Pure COPD	3,186 (50.47)	1.169	1.155	1.183	1.116	1.100	1.131
ACO	3,127 (49.53)	1.163	1.149	1.177	1.124	1.109	1.140
**1 month**							
Total COPD	6,313	1.035	1.019	1.051	1.022	1.009	1.036
Pure COPD	3,186 (50.47)	1.035	1.012	1.059	1.017	0.998	1.036
ACO	3,127 (49.53)	1.035	1.012	1.057	1.026	1.007	1.046

COPD = chronic obstructive pulmonary disease, ACO = asthma and COPD overlap, HR = hazard ratio, CI = confidence interval.

*Adjusted by sex, age, Charlson Comorbidity Index, smoking status, body mass index, and household income level.

### Subgroup analysis of non-accidental mortality

In the mortality analysis, we found that sex and smoking status were interacted with PM_10_ concentrations, respectively (P = 0.0001 for sex, P = 0.0525 for never smoker *vs*. once smoker, and P = 0.1367 for never smoker *vs*. former smoker *vs*. current smoker, respectively). Therefore, stratified analysis was performed by sex and smoking status. The mortality for PM_10_ exposure was analyzed for each group (pure COPD and ACO) according to each period (1, 3, 6 and 12 months), and aHRs for each period showed similar patterns for each group (Table 3). Subgroup analysis also showed that the effect of average exposure over 3 months was the most significant among the groups.

**Table 3:** Hazard ratios for death stratified by sex according to PM_10_ exposure duration.

	N (%)	HR	95% CI		aHR	95% CI	
**12 months**							
**Men**							
Total COPD	3,386	1.006	0.987	1.025	1.000	0.981	1.019
Pure COPD	1,763 (55.34)	1.002	0.976	1.029	0.992	0.965	1.019
ACO	1,623 (51.90)	1.009	0.982	1.037	1.005	0.978	1.033
**Women**							
Total COPD	2,927	1.044	1.011	1.079	1.048	1.014	1.082
Pure COPD	1,423 (44.66)	1.047	0.995	1.102	1.050	0.994	1.109
ACO	1,504 (48.10)	1.041	0.999	1.086	1.037	0.995	1.082
**6 months**							
**Men**							
Total COPD	3,386	1.161	1.148	1.174	1.118	1.104	1.132
Pure COPD	1,763 (55.34)	1.166	1.148	1.183	1.121	1.101	1.141
ACO	1,623 (51.90)	1.156	1.137	1.175	1.119	1.098	1.141
**Women**							
Total COPD	2,927	1.221	1.195	1.248	1.138	1.112	1.165
Pure COPD	1,423 (44.66)	1.255	1.209	1.304	1.132	1.085	1.181
ACO	1,504 (48.10)	1.203	1.170	1.236	1.143	1.107	1.180
**3 months**							
**Men**							
Total COPD	3,386	1.150	1.139	1.161	1.112	1.100	1.124
Pure COPD	1,763 (55.34)	1.153	1.138	1.167	1.115	1.097	1.132
ACO	1,623 (51.90)	1.148	1.131	1.164	1.116	1.097	1.135
**Women**							
Total COPD	2,927	1.201	1.180	1.223	1.144	1.119	1.169
Pure COPD	1,423 (44.66)	1.215	1.177	1.254	1.124	1.081	1.169
ACO	1,504 (48.10)	1.194	1.168	1.220	1.153	1.121	1.185
**1 month**							
**Men**							
Total COPD	3,386	1.020	1.002	1.039	1.014	0.999	1.030
Pure COPD	1,763 (55.34)	1.033	1.008	1.058	1.017	0.996	1.038
ACO	1,623 (51.90)	1.006	0.980	1.033	1.009	0.985	1.033
**Women**							
Total COPD	2,927	1.071	1.038	1.105	1.046	1.020	1.073
Pure COPD	1,423 (44.66)	1.021	0.967	1.079	1.013	0.971	1.056
ACO	1,504 (48.10)	1.095	1.056	1.134	1.060	1.029	1.091

COPD = chronic obstructive pulmonary disease, ACO = asthma and COPD overlap, HR = hazard ratio, CI = confidence interval.

*Adjusted by age, Charlson Comorbidity Index, smoking status, body mass index, and household income level.

**Figure 4:**
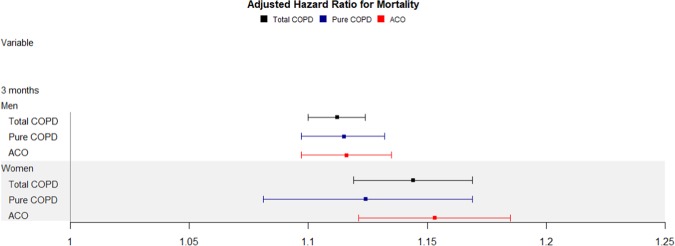
Effect of particular matter on mortality stratified by sex over a 3-month PM_10_ exposure. COPD = chronic obstructive lung disease, ACO = asthma-COPD overlap.

**Figure 5:**
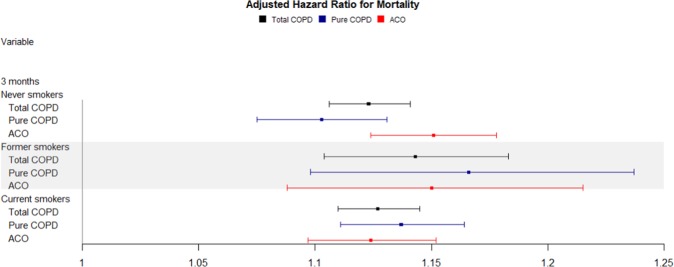
Effect of particular matter on mortality stratified by smoking status over a 3-month PM_10_ exposure. COPD = chronic obstructive lung disease, ACO = asthma-COPD overlap.

In women, the aHRs for each 10-μg/m^3^ increment in PM_10_ after a 3-month average exposure were 1.124 (95% CI, 1.081–1.169) for pure COPD, and 1.153 (95% CI, 1.121–1.185) for ACO (Table 3 and Fig. 4). When aHRs were analyzed in stratified subgroups according to smoking habit, the results were 1.103 (95% CI, 1.075–1.131) for pure COPD and 1.151(95% CI, 1.124–1.178) for ACO in never smokers. However, in former smokers, they were 1.166 (95% CI, 1.098–1.237) for pure COPD and 1.150 (95% CI, 1.088–1.215) for ACO. In current smokers, aHRs were 1.137 (95% CI, 1.111–1.164) for pure COPD and 1.124 (95% CI, 1.097–1.152) for ACO (Tables 4–7 and Fig. 5). Additionally, in once smokers including former and current smokers, aHRs were 1.136 (95% CI, 1.114–1.159) for pure COPD and 1.116 (95% CI, 1.095–1.138) for ACO (Supplementary Table S1 and Fig. S1).

**Table 4:** Hazard ratios for death stratified by smoking status according to PM_10_ exposure duration (12 months).

	N	HR	95% CI		aHR	95% CI	
**12 months**							
**Never smokers**							
Total COPD	3,348	1.027	1.001	1.054	1.026	1.001	1.053
Pure COPD	1,678 (52.67)	1.037	1.000	1.076	1.031	0.991	1.072
ACO	1,670 (53.41)	1.018	0.983	1.054	1.021	0.987	1.057
**Former smokers**							
Total COPD	967	0.992	0.951	1.035	0.988	0.945	1.033
Pure COPD	502 (15.76)	0.984	0.921	1.051	0.985	0.917	1.058
ACO	465 (14.87)	0.996	0.942	1.052	0.980	0.923	1.040
**Current smokers**							
Total COPD	1,998	1.015	0.989	1.041	1.006	0.981	1.032
Pure COPD	1,006 (31.58)	1.000	0.965	1.036	0.981	0.946	1.017
ACO	992 (31.72)	1.031	0.994	1.069	1.028	0.991	1.067

COPD = chronic obstructive pulmonary disease, ACO = asthma-COPD overlap, HR = hazard ratio, CI = confidence interval.

*Adjusted by sex, age, Charlson Comorbidity Index, body mass index, and household income level.

**Table 5:** Hazard ratios for death stratified by smoking status according to PM_10_ exposure duration (6 months).

	N	HR	95% CI		aHR	95% CI	
**6 months**							
**Never smokers**							
Total COPD	3,348	1.197	1.178	1.217	1.134	1.114	1.154
Pure COPD	1,678 (52.67)	1.205	1.177	1.234	1.137	1.103	1.171
ACO	1,670 (53.41)	1.189	1.162	1.216	1.141	1.112	1.171
**Former smokers**							
Total COPD	967	1.189	1.156	1.223	1.145	1.105	1.186
Pure COPD	502 (15.76)	1.191	1.146	1.236	1.193	1.139	1.249
ACO	465 (14.87)	1.152	1.081	1.228	1.153	1.095	1.214
**Current smokers**							
Total COPD	1,998	1.160	1.143	1.176	1.127	1.107	1.148
Pure COPD	1,006 (31.58)	1.166	1.144	1.189	1.127	1.099	1.155
ACO	992 (31.72)	1.152	1.128	1.176	1.133	1.101	1.166

COPD = chronic obstructive pulmonary disease, ACO = asthma-COPD overlap, HR = hazard ratio, CI = confidence interval.

*Adjusted by sex, age, Charlson Comorbidity Index, body mass index, and household income level.

**Table 6:** Hazard ratios for death stratified by smoking status according to PM_10_ exposure duration (3 months).

	N	HR	95% CI		aHR	95% CI	
**3 months**							
**Never smokers**							
Total COPD	3,348	1.176	1.160	1.192	1.123	1.106	1.141
Pure COPD	1,678 (52.67)	1.171	1.148	1.194	1.103	1.075	1.131
ACO	1,670 (53.41)	1.180	1.158	1.202	1.151	1.124	1.178
**Former smokers**							
Total COPD	967	1.165	1.138	1.192	1.143	1.104	1.183
Pure COPD	502 (15.76)	1.169	1.135	1.204	1.166	1.098	1.237
ACO	465 (14.87)	1.174	1.122	1.229	1.150	1.088	1.215
**Current smokers**							
Total COPD	1,998	1.155	1.141	1.169	1.127	1.110	1.145
Pure COPD	1,006 (31.58)	1.161	1.142	1.182	1.137	1.111	1.164
ACO	992 (31.72)	1.148	1.128	1.168	1.124	1.097	1.152

COPD = chronic obstructive pulmonary disease, ACO = asthma-COPD overlap, HR = hazard ratio, CI = confidence interval.

*Adjusted by sex, age, Charlson Comorbidity Index, body mass index, and household income level.

**Table 7:** Hazard ratios for death stratified by smoking status according to PM_10_ exposure duration (1 month).

	N	HR	95% CI		aHR	95% CI	
**1 month**							
**Never smokers**							
Total COPD	3,348	1.046	1.021	1.072	1.028	1.009	1.049
Pure COPD	1,678 (52.67)	1.040	1.002	1.079	1.012	0.984	1.041
ACO	1,670 (53.41)	1.051	1.017	1.086	1.040	1.014	1.068
**Former smokers**							
Total COPD	967	0.981	0.940	1.023	0.999	0.963	1.036
Pure COPD	502 (15.76)	1.070	1.014	1.129	1.045	0.997	1.095
ACO	465 (14.87)	0.908	0.859	0.960	0.940	0.893	0.988
**Current smokers**							
Total COPD	1,998	1.040	1.016	1.065	1.025	1.004	1.046
Pure COPD	1,006 (31.58)	1.015	0.980	1.050	1.006	0.977	1.036
ACO	992 (31.72)	1.062	1.031	1.094	1.039	1.011	1.068

COPD = chronic obstructive pulmonary disease, ACO = asthma-COPD overlap, HR = hazard ratio, CI = confidence interval.

*Adjusted by sex, age, Charlson Comorbidity Index, body mass index, and household income level.

## Discussion

In this national study, we found that average exposure to PM_10_ at various periods (1, 3, 6 or 12 months) increased the risk of death in patients with COPD, particularly ACO. When stratifying the total COPD group, mortality according to the concentration of PM10 in the ACO group was greater than that of PM10 in pure COPD. Since this is not a direct comparison between the two groups (pure COPD vs. ACO), it cannot be concluded that the ACO patients has a higher mortality rate than pure COPD. However, death for ACO patients can be said to be more affected by PM10 under all exposure periods. Six month of exposure had the most significant impact on mortality. However, the difference in mortality between pure COPD patients and ACO patients was the greatest during the 3-month exposure period. A comprehensive analysis based on 3-month exposure was based on stratification according to sex and smoking status. Higher aHRs were found in women in the ACO group and the never-smoker ACO group. However, among smokers, patients with pure COPD manifested a higher risk of death compared with patients with ACO. Among smokers, the risk of death was higher in former smokers than in current smokers.

Previous epidemiologic studies have suggested that PM_10_ affects acute or chronic COPD mortality. A systematic review and meta-analysis revealed that outdoor air pollution increases morbidity and mortality in COPD patients^14^. In this study, acute exposure was associated with increased COPD mortality and chronic PM exposure resulted in a 10% increase in mortality. Also, a mortality study^15^ in COPD patients was conducted over an average period of 12-months of PM_10_ exposure based on hospital data in 34 US cities. The study revealed significant associations, with a HR of 1.11 (95% CI: 1.06–1.15) for 10-μg/m^3^ increase in PM_10_ during the same year. In the distributed lag model, the sum of PM exposure effects during the four years (same year and 3 preceding years) showed a HR for mortality of 1.22 (95% CI: 1.17–1.27). The mortality for a 1-year average exposure reported in this study differed from our study results, probably due to the differences in the methods used to calculate the mean PM exposure. In their study, all of the annual mean concentrations of the same year were included similar to those exposed to death, but our study used, only the average exposure from the day of the event until a year ago. The effect of PM_10_ on total mortality in COPD patients was evaluated in a number of studies investigating short-term exposure, which mainly assessed daily exposure (within 7 days of the lag day)^16–18^. Very few studies investigated the long-term exposure, involving an average exposure for 1 to 4 years^19,20^. However, this study reported the effects of average exposure at 1, 3, 6, and 12 months, to evaluated the effects of short-term and long-term exposure, continuously.

In this study, the effect of PM_10_ concentration on mortality in ACO and COPD patients was not statistically significant following long-term exposure (12 months), but was statistically significant during short-term exposure (1,3, and 6 months), respectively. This effect may be attributed to the existence of several confounding factors such as seasonality during the period to account for the relationship between the average concentration of PM and deaths among the subjects in 1 year. However, aHRs was statistically significant during the one-month exposure period only in the ACO group, which is likely to have been more sensitive to recent PM_10_ exposures because ACO patients exhibit airway hyper-responsiveness compared with pure COPD patients. Further, a study of the natural history of COPD suggested that mortality peaks during the 3-month period following the onset of severe exacerbation^21^. Similarly, our study showed a statistically significant increase in mortality following average exposure over a 3-month period. Thus, in order for PM_10_ to influence the mortality associated with COPD, the increase in mortality may be strongly associated with statistically significant exposure over the specific time period.

Although epidemiological studies have suggested that PM affects the mortality of COPD patients, in-depth epidemiological outcomes in patients with ACO have yet to be reported. ACO was defined by a joint project of the Global Initiative for Asthma (GINA) committee and the Global Initiative for Chronic Obstructive Lung Disease (GOLD) committee in 2014^4^. ACO patients tend to manifest respiratory symptoms more frequently than those diagnosed with pure COPD probably due to airway hyper-responsiveness^6,22^. Therefore, the mortality associated with PM_10_ exposure is also expected to differ between patients with pure COPD and ACO. In the Danish cohorts (Diet, Cancer, and Health cohort, 1993–1997), the total mortality was higher among participants with ACO (25.9 per 1,000 person-years) than in those with asthma or COPD alone (7.9 [*P* < 0.001] and 23.1 [*P* < 0.01] per 1,000 person-years, respectively)^23^.

The recent increase in PM concentration due to environmental pollution can aggravate symptoms more often in respiratory patients. Dyspnea reduces daily activity in patients and can cause muscle weakness and contraction, including respiratory muscles, which in turn induces breathing difficulties, resulting in increased mortality^24^. This study showed that women with ACO showed a high risk of death associated with PM_10_ exposure, which suggests that physiological dynamics in women, such as relatively low muscle mass may contribute to the vicious cycle.

In terms of smoking status, the PM10 effect on mortality was observed to be greatest among former smokers compared with never and current smokers. These findings can be misleading suggesting that smoking cessation has a negative effect. In fact, former smokers often quit smoking due to health problems. Therefore, they are likely to be vulnerable to smoking and at a high risk for smoking-related illness or death^25–27^. Conversely, current smokers are likely to be relatively resistant to the harmful effect of smoking, often called healthy smoker effect. In addition, cigarette smoking may contribute more to centrilobular emphysema and less to airway remodeling, and the smoke biomass mainly causes airway remodeling rather than emphysema^28^. These results suggest that the effect of PM on lung function may differ from the effects of smoking. Therefore, the relationship between smoking status and the effect of PM on ACO and pure COPD is complex.

Our study was the first of its kind conducted to examine the mortality risk associated with PM_10_ exposure in patients with ACO and pure COPD. In addition, the CCI reflected the health status affecting mortality. Nevertheless, this study has several limitations. Data on PM_2.5_, which was recently identified as an important factor affecting mortality with PM, could not be used for analysis because they were not gathered during the study period. Although mortality due to PM_10_ in patients with COPD may be attributed to several other factors, the study established the most simplified model. Therefore, we will analyze the effects of CVD, lung cancer, and other conditions, which play a major role, in future studies. Further, COPD and ACO diagnoses were established according to ICD-10 and prescriptions, and not based on clinical diagnosis. Nevertheless, these COPD and ACO definitions are commonly used in other studies based on health insurance claims data not only in Korea but also in Taiwan and the United States, and these studies might reflect the real-world scenario including health care utilization. In addition, our study is more valuable than the other studies using these data because we used precise information such as household income, smoking history, and body mass index from biennial health screenings in our analysis.

## Conclusion

Average exposure to PM_10_ was associated with non-accidental mortality in patients with COPD, especially ACO. In addition, the adverse effects of PM_10_ exposure on non-accidental death in ACO were greater among women and in never-smokers. It is imperative to evaluate the high-risk groups and the underlying physiological mechanisms associated with the impact of air pollution on health. Since PM exposure has been reported to induce reactive oxygen species production and inflammation in the respiratory system, it is necessary to determine whether nutrient supplements such as antioxidants can actually reduce individual’ susceptibility to air pollution in COPD patients. Thus, in accordance with the precautionary principle, these results can be used as a basis for a policy to reduce the emissions of air pollution in order to improve the respiratory health of individuals.

## Supplementary information

Supplementary Information.

## Data Availability

The data that support the findings of this study are available from the National Health Insurance Service–National Sample Cohort (NHIS-NSC) but restrictions apply to the availability of these data, which were used under license for the current study, and so are not publicly available. Data are however available from the authors upon reasonable request and with permission of the National Health Insurance Service–National Sample Cohort (NHIS-NSC).
