# Supplementary material for: Effects of PM10 on mortality in pure COPD and asthma-COPD overlap: difference in exposure duration, gender, and smoking status
Source: Sci Rep. 2020 Feb 12;10:2402. doi: 10.1038/s41598-020-59246-2 (PMC7016150; doi:10.1038/s41598-020-59246-2)

Effects of PM_{10} on mortality in pure COPD and asthma-COPD overlap: difference in exposure duration, gender, and smoking status.

Author list

Yu Min Lee[1*] Jin Hwa Lee[2], Hawn-Cheol Kim[3] Eun Hee Ha[1†]

Author Affiliations

[1]Department of Occupational and Environmental Medicine, Ewha Womans University College of Medicine, Seoul, Republic of Korea.

[2]Division of Pulmonary and Critical Care Medicine, Department of Internal Medicine, Ewha Womans University College of Medicine, Seoul, Republic of Korea.

[3]Department of Occupational and Environmental Medicine, Inha University School of Medicine, Incheon, Republic of Korea

†**Corresponding authors:** Eun Hee Ha, Department of Occupational and Environmental Medicine, Ewha Womans University College of Medicine, 25, Magokdong-ro 2-gil, Gangseo-gu, Seoul, Republic of Korea (07804), E-mail: eunheeha@ewha.ac.kr

Competing interests

The author(s) declare no competing interests..

Supplementary Table S1. Hazard Ratio for death stratified by smoking status (binary) to PM_{10} exposure duration.

	N	HR	95% CI		aHR	95% CI	
**12 months**							
**Never smokers**							
**Total COPD**	3,348	1.027	1.001	1.054	1.026	1.001	1.053
**Pure COPD**	1,678	1.037	1.000	1.076	1.031	0.991	1.072
**ACO**	1,670	1.018	0.983	1.054	1.021	0.987	1.057
**Once smokers**							
**Total COPD**	2,965	1.009	0.987	1.031	1.004	0.982	1.025
**Pure COPD**	1,508	0.997	0.967	1.029	1.020	0.989	1.051
**ACO**	1,457	0.990	0.960	1.022	1.014	0.983	1.045
**6 months**							
**Never smokers**							
**Total COPD**	3,348	1.197	1.178	1.217	1.134	1.114	1.154
**Pure COPD**	1,678	1.205	1.177	1.234	1.137	1.103	1.171
**ACO**	1,670	1.189	1.162	1.216	1.141	1.112	1.171
**Once smokers**							
**Total COPD**	2,965	1.165	1.151	1.180	1.125	1.108	1.142
**Pure COPD**	1,508	1.172	1.152	1.192	1.131	1.107	1.156
**ACO**	1,457	1.158	1.137	1.179	1.124	1.098	1.150
**3 months**							
**Never smokers**							
**Total COPD**	3,348	1.176	1.160	1.192	1.123	1.106	1.141
**Pure COPD**	1,678	1.171	1.148	1.194	1.103	1.075	1.131
**ACO**	1,670	1.180	1.158	1.202	1.151	1.124	1.178
**Once smokers**							
**Total COPD**	2,965	1.158	1.145	1.170	1.122	1.107	1.136
**Pure COPD**	1,508	1.164	1.147	1.182	1.136	1.114	1.159
**ACO**	1,457	1.150	1.132	1.169	1.116	1.095	1.138
**1 month**							
**Never smokers**							
**Total COPD**	3,348	1.046	1.021	1.072	1.028	1.009	1.049
**Pure COPD**	1,678	1.040	1.002	1.079	1.012	0.984	1.041
**ACO**	1,670	1.051	1.017	1.086	1.040	1.014	1.068
**Once smokers**							
**Total COPD**	2,965	1.025	1.004	1.046	1.021	1.002	1.039
**Pure COPD**	1,508	1.030	1.000	1.060	1.022	0.997	1.048
**ACO**	1,457	1.020	0.991	1.051	1.016	0.990	1.042

COPD = chronic obstructive pulmonary disease, ACO = asthma-COPD overlap, HR = hazard ratio, CI = confidence interval

*Adjusted by age, Charlson Comorbidity Index, smoking status, body mass index, and household income level.

Supplementary figure S1.

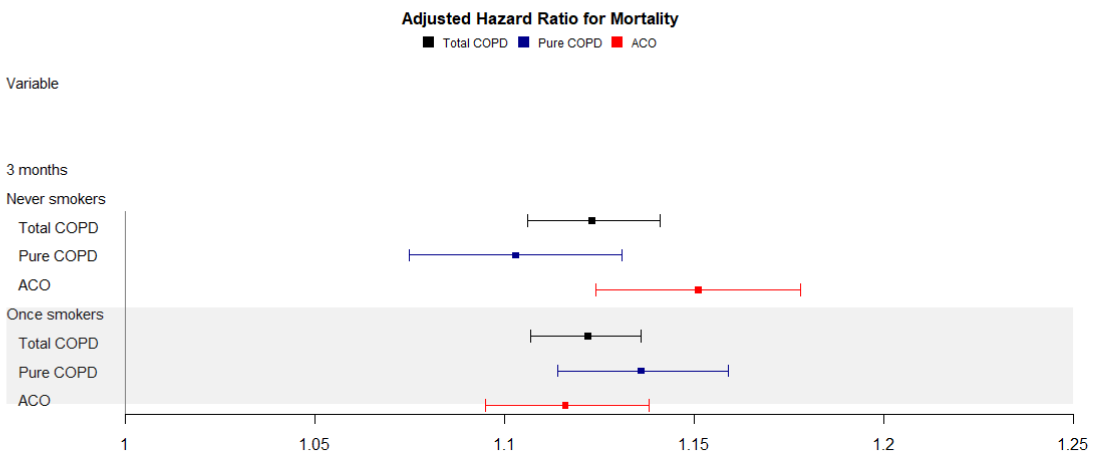

Supplement: Supplementary file 1 — Supplementary Information. [file 41598_2020_59246_MOESM1_ESM.pdf]
